# Circadian rhythm disruption declines oocyte quality for fertility via PTEN/AKT pathway

**DOI:** 10.1093/procel/pwaf080

**Published:** 2025-09-24

**Authors:** Ping-Shuang Lu, Kun-Huan Zhang, Si-Le Wu, Rui-Jie Ma, Yuan-Jing Zou, Jia-Qian Ju, Hao-Lin Zhang, Yue Wang, Shao-Chen Sun

**Affiliations:** College of Animal Science and Technology, Nanjing Agricultural University, Nanjing 210095, China; College of Animal Science and Technology, Nanjing Agricultural University, Nanjing 210095, China; College of Animal Science and Technology, Nanjing Agricultural University, Nanjing 210095, China; College of Animal Science and Technology, Nanjing Agricultural University, Nanjing 210095, China; College of Animal Science and Technology, Nanjing Agricultural University, Nanjing 210095, China; College of Animal Science and Technology, Nanjing Agricultural University, Nanjing 210095, China; College of Animal Science and Technology, Nanjing Agricultural University, Nanjing 210095, China; College of Animal Science and Technology, Nanjing Agricultural University, Nanjing 210095, China; College of Animal Science and Technology, Nanjing Agricultural University, Nanjing 210095, China; Key Laboratory of Research on Clinical Molecular Diagnosis for High Incidence Diseases in Western Guangxi of Guangxi Higher Education Institutions, Reproductive Medicine of Guangxi Medical and Health Key Discipline Construction Project, Affiliated Hospital of Youjiang Medical University for Nationalities, Baise 533000, China

**Dear Editor,**

Circadian rhythm is characterized by the synchronization of circadian clocks throughout the body, driven by the external light/dark cycle. Artificial light sources and irregular lifestyles can disrupt biological rhythms, leading to metabolic syndrome, brain injuries, and inflammatory responses (Taillard et al., 2021). In humans, reproduction is affected by artificial light pollution, showing increased risk of polycystic ovary syndrome (PCOS), prevalence of menstrual cycle irregularity, and adverse pregnancy outcomes (Cai et al., 2019). In animal experiments, decreased ovarian luteal function was reported after mice were continuously exposed to 24 h light for more than 6 weeks (Li et al., 2023), while short period of illumination with 12 days led to reduced mouse ovarian follicle numbers accompanied by increased cell apoptosis and DNA damage (Li et al., 2019). Although the negative effects of circadian disruption on female reproduction were reported, the effects on oocyte quality, especially the related molecular mechanisms have not been well investigated.

Oocyte maturation is a prerequisite for fertilization and embryo development. Mitochondria are the main organelles in the cell that play various roles in energy production, calcium homeostasis, and materials exchanges through their connections with other organelles such as the endoplasmic reticulum (ER) and Golgi apparatus (Kirillova et al., 2021). The imbalance of mitochondria has been found to associate with the pathogenesis of premature ovarian insufficiency, reproductive aging, and obesity-related adverse pregnancy outcome in female mammals (Zhang et al., 2023; Cochrane et al., 2024; Shang et al., 2025). Given the important regulatory roles of mitochondria in females, we sought to determine whether mitochondrial dysfunction contributes to adverse reproductive outcomes caused by circadian disruption.

In this study, we established a circadian rhythm disruption mouse model. Melatonin, an important circadian rhythm regulator, was simultaneously administered in the drinking water for rescue treatment (named light + MT) at a concentration of 30 mg/kg BW, based on a previous study (Lan et al., 2018) (Fig. 1A). No significant difference in body weight was found (Fig. S1A); however, significantly irregular and prolonged estrous cycle were confirmed in light-exposed mice (Fig. S1B and S1C). Fertility tests showed a significant reduction in pup number and survival rate in light exposure groups (Figs. 1B and S1D), with no difference in offspring body weight (Fig. S1E). The ovary/body weight ratio and oocyte numbers per mouse also showed no significant differences among the control, light, and light + MT groups (Fig. S1F and S1G). Whereas, the circadian-related mRNA levels of *Csnk1e* and *Bmal1* in MI stage oocytes were significantly changed in light-exposed mice (Fig. S1H). Subsequent analysis revealed that circadian disruption did not affect meiosis resumption, but significantly reduced first oocyte polar body (PB1) extrusion and zygote formation (Figs. 1C, 1D and S1I). These results demonstrated that constant light exposure induced mouse fertility decline with abnormal estrus cycle and reduced oocyte maturation capacity, which could be ameliorated by melatonin supplementation.

**Figure 1.**
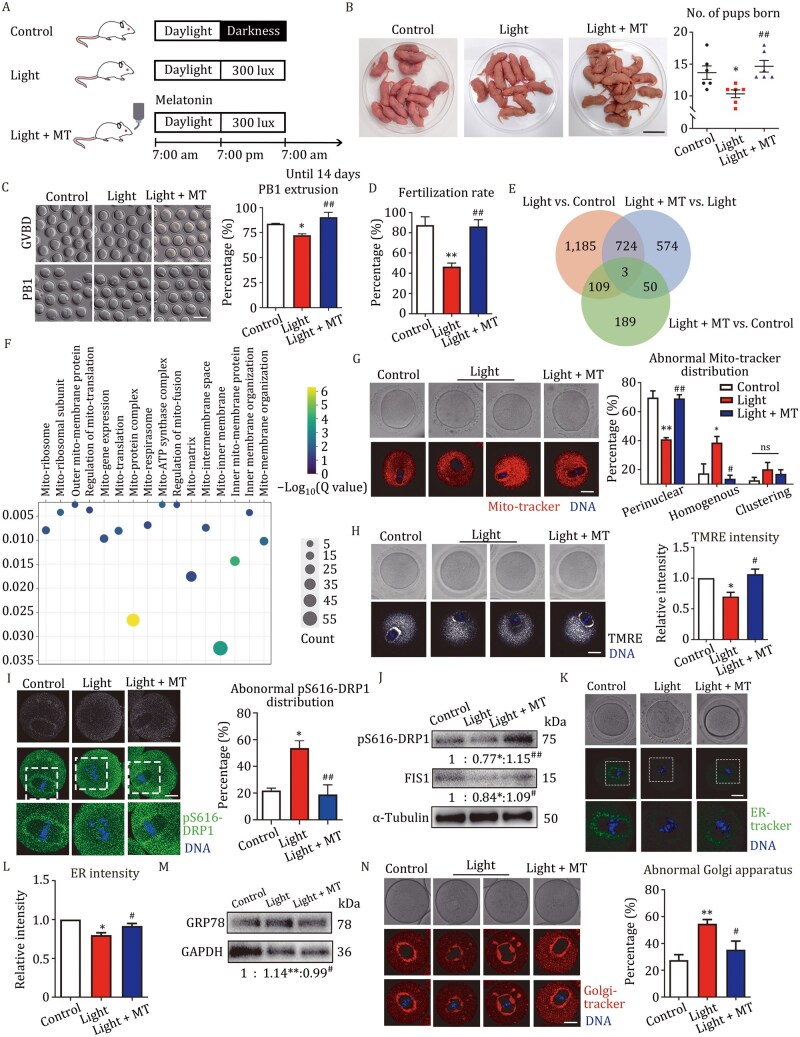
**Constant light exposure affects oocyte mitochondrial functions for fertility**. (A) Diagram and timeline of illumination pattern and melatonin administration in mice exposed to constant light. (B) Representative images of pups delivered in the control, light, and light + MT mice. Bar = 3 cm. The number of pups born in the control (*n *= 6), light (*n *= 6), and light + MT (*n *= 6) female mice was quantified after mating with male mice. (C) Representative images of oocytes that underwent GVBD after 2 h culture and PB1 extrusion after 12 h culture in the control, light, and light + MT mice. Bar = 80 μm. The percentage of PB1 extrusion in the control (*n *= 116), light (*n *= 145), and light + MT (*n *= 148) mice oocytes was quantified. (D) The fertilization rate in the control (*n *= 119), light (*n *= 129), and light + MT (*n *= 124) group. (E) Venn diagram of DEGs in control, light, and light + MT oocytes of MI stage. (F) The bubble graph of mitochondria-related GO pathways based on the DEGs between the control and light groups. (G) Representative images and different distribution patterns of mitochondria in oocyte of control (*n *= 45), light (*n *= 49), and light + MT (*n *= 42) groups. Bar = 20 μm. (H) Representative images and relative intensity of mitochondria membrane potential in oocyte of the control (*n *= 34), light (*n *= 35), and light + MT (*n *= 35) groups using TMRE staining. Bar = 20 μm. (I) Representative images and abnormal distribution of pSer616-DRP1 in oocyte of the control (*n *= 46), light (*n *= 51), and light + MT (*n *= 46) groups. Bar = 20 μm. (J) Band intensity analysis of pSer616-DRP1 and FIS1 in the MI stage oocytes of control, light, and light + MT groups. (K) Representative images of ER-tracker in oocyte of control, light, and light + MT groups. Bar = 20 μm. (L) Relative intensity of ER-tracker in oocyte of control (*n *= 44), light (*n *= 40), and light + MT (*n *= 38) groups. (M) Band intensity analysis of GRP78 in MI stage oocytes of control, light, and light + MT groups. (N) Representative images and abnormal percentage of Golgi apparatus in oocyte from control (*n *= 60), light (*n *= 65), and light + MT (*n *= 55) groups. Bar = 20 μm. *indicates the significance between the control and light groups. ^#^indicates the significance between the light and light + MT groups. Data are presented as mean ± SEM of three biological repeats. ns, *P *> 0.05; *P < 0.05; **P < 0.01; ^#^*P *< 0.05; ^##^*P *< 0.01.

To explore the potential mechanisms underlying the decreased oocyte quality from light-exposed mice, RNA-seq was performed. There were 2021 differentially expressed genes (DEGs) in the light exposure groups compared with control groups, while apparently reduced DEGs were found between the control and light + MT groups (Figs. 1E and S2A). Analysis of Gene Ontology (GO) and Kyoto Encyclopedia of Genes and Genomes (KEGG) displayed that the DEGs were significantly enriched in protein processing in the ER, microtubule organization, and organelle fission (Fig. S2B–F), especially in mitochondrial compartments (Figs. 1F and S2G). Despite the importance of mRNA stability in regulating RNA storage, few DEGs were enriched in pathways related to RNA stability regulation. Further analysis revealed significantly increased abnormal mitochondrial distribution in light-exposed oocytes, with most exhibiting homogeneous or clustering patterns (Fig. 1G). Moreover, light-exposed oocytes showed decreased mitochondrial membrane potential, reduced mitochondrial DNA copy number, and lower ATP content (Figs. 1H, S3A and S3B). To investigate impaired mitochondria dynamics, we examined mitochondria fission-related proteins and found significantly diminished spindle periphery localization of pSer616-DRP1 and reduced pS616-DRP1/FIS1 expression in light-exposed oocytes (Fig. 1I and 1J). Given the close communication between mitochondria and other organelles, and GO analysis that highlighted DEGs linked to organelles dynamics (Fig. S3C), we also investigated other key organelles. We observed weaker ER signals at the spindle ­periphery and elevated GRP78 levels in light-exposed oocytes, indicating the occurrence of ER stress (Figs. 1K–M and S4A). Golgi apparatus staining revealed either scattered or strongly clustered distribution patterns in the cytoplasm of light-exposed oocyte (Fig. 1N). Furthermore, Rab10 and Rab11a signals were obviously aggregated in the subcortical region, with significantly increased cytoplasmic intensity following light exposure (Fig. S4B and S4C). Meanwhile, the rate of cortical granule lacking a cortical granule-free domain (CGFD) was dramatically increased after circadian disruption (Fig. S4D). These results indicated that constant light exposure disrupted organelle dynamics and functions in mouse oocytes. We then employed the mitochondria-­targeted antioxidant mitoquinone (MitoQ) to determine whether mitochondrial dysfunction was a key mechanism underlying the light exposure effects. Since the close association between mitochondrial function and cellular redox homeostasis, we measured the reactive oxygen species (ROS) level. Treatment with 0.1 μmol/L MitoQ significantly reduced abnormal ROS levels in light-exposed oocytes *in vitro* (Fig. S3D). Additionally, MitoQ supplementation restored the ATP levels and normal Golgi apparatus distribution patterns at the spindle periphery in light-exposed oocytes (Fig. S3E and S3F). These results suggested that mitochondria-targeted MitoQ ameliorated mitochondria energy metabolism and Golgi apparatus distribution in light-exposed mouse oocyte.

Next, we investigated how circadian disruption induced oocyte mitochondrial dysfunction. KEGG analysis revealed significant enrichment of DEGs in the AKT signaling pathway (Fig. S2D). Gene Set Enrichment Analysis (GSEA) illustrated the downregulated AKT activity (Fig. 2A). Western blot and fluorescence staining results confirmed this, showing significantly reduced levels of pSer473-AKT and AKT, along with increased expression of phosphatases and tension homolog (PTEN), a negative regulator of AKT, in light-exposed oocytes (Fig. 2B and 2C). Treatment with the AKT-specific agonist SC79 rescued the defects in PB1 extrusion, cellular ROS levels, mitochondrial spindle periphery distribution, and ATP production (Figs. 2D–F and S3E). Based on DEGs related to cytoskeleton organization, we next evaluated spindle formation and actin assembly. Oocytes from constant light-exposed mice displayed aberrant spindle, whereas SC79 treatment restored a typical barrel-shaped spindle with focused poles (Fig. 2G). F-actin labelling revealed decreased cortical and cytoplasmic signals in light-exposed oocytes, which were rescued by SC79 treatments (Fig. 2H and 2I). These results demonstrated that AKT activator SC79 ameliorated oocyte meiotic defects caused by circadian disruption through restoration of mitochondrial functions and cytoskeletal dynamics.

**Figure 2.**
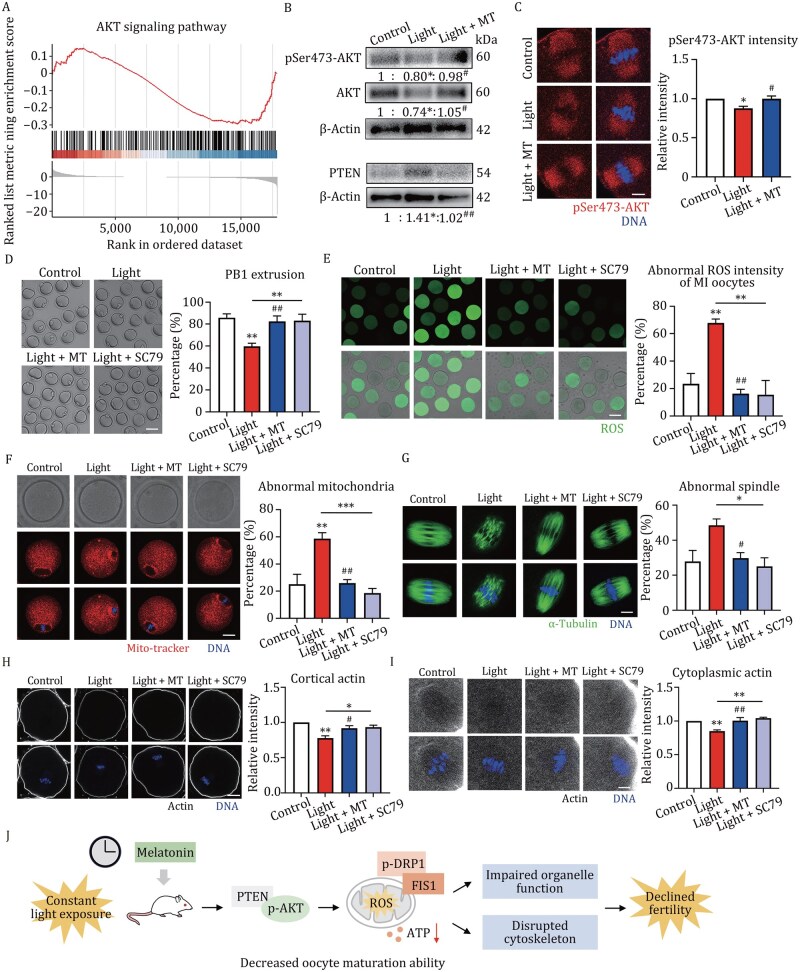
**AKT activator SC79 ameliorates oocyte meiotic defects caused by circadian disruption**. (A) Gene Set Enrichment Analysis (GSEA) based on the normalized FPKMs of all genes detected in the light exposure groups. (B) Band intensity analysis of AKT, pS473-AKT, PTEN in MI stage oocytes of control, light, and light + MT groups. (C) Representative images and relative intensity of pS473-AKT in MI stage oocyte from control (*n *= 48), light (*n *= 47), and light + MT (*n *= 50) groups. Bar = 10 μm. (D) Representative images and percentage of PB1 extrusion after 12 h culture in control (*n *= 123), light (*n *= 112), light + MT (*n *= 134), and light + SC79 (*n *= 123) mouse oocytes. Bar = 80 μm. (E) Representative images and abnormal intensity of ROS level in MI stage oocyte from control (*n *= 69), light (*n *= 50), light + MT (*n *= 55), and light + SC79 (*n *= 52) mice. Bar = 80 μm. The intensity of ROS that was above the mean + SEM was considered abnormal. (F) Representative images and abnormal distribution of mitochondria in MI stage oocyte of control (*n *= 46), light (*n *= 51), light + MT (*n *= 47), and light + SC79 (*n *= 48) mice. Homogeneous or clusters of mitochondrial distribution patterns in the cytoplasm were considered abnormal. Bar = 20 μm. (G) Representative images and abnormal spindle in control (*n *= 45), light (*n *= 37), light + MT (*n *= 40), and light + SC79 (*n *= 43) mice. Bar = 10 μm. (H) Representative images and relative intensity of cortical actin filaments in MI stage oocyte of control (*n *= 40), light (*n *= 31), light + MT (*n *= 40), and light + SC79 (*n *= 53) mice. Bar = 20 μm. (I) Representative images and relative intensity of cytoplasmic actin filaments in MI stage oocyte of control (*n *= 40), light (*n *= 31), light + MT (*n *= 40), and light + SC79 (*n *= 53) mice. Bar = 10 μm. (J) Schematic diagram illustrating the effects and mechanisms of circadian rhythm disruption by constant light exposure on fertility. Constant light exposure inhibited mitochondrial energy production and FIS1/pSer616-DRP1-related dynamics via the PTEN/AKT pathway, disrupting organelle function and cytoskeletal organization essential for oocyte maturation. These alterations ultimately led to declined fertility. Melatonin administration could alleviate these defects in oocytes from mice exposed to constant light. *indicates the significance between the control, light, and light + SC79 groups. ^#^indicates the significance between the light and light + MT groups. Data are presented as mean ± SEM of three biological repeats. *P < 0.05. **P < 0.01. ***P < 0.001. ^#^*P *< 0.05. ^##^*P *< 0.01.

In this study, for the first time, we investigated the negative effects and molecular mechanism of circadian rhythm changes on female reproduction from oocyte quality aspect. We found that constant light exposure led to declined mouse fertility with abnormal estrus cycle, which could be due to reduced oocyte quality caused by mitochondrial dysfunction via the PTEN/AKT pathway, and these defects could be restored by the melatonin supplementation (Fig. 2J).

Due to ever-changing work and artificial light at night, a growing number of studies have illustrated that rhythm changes impair female reproduction (Cai et al., 2019; Li et al., 2019; Li et al., 2023). However, the molecular mechanisms of circadian disruption are still unclear. Using the circadian rhythm disruption mouse model, we first reported that light exposure reduced pup production and offspring survival rate, which may be due to impaired oocyte quality. Previous studies also reported influenced ovarian luteinization in light-exposed mice (Li et al., 2023) and impaired oocyte meiosis progression in Bmal1- or Casein kinase 1α-deficient mice (Zhang et al., 2022), indicating the close interaction between circadian rhythm and female reproduction. To elucidate the mechanisms underlying light exposure-induced oocyte defects, we performed RNA-seq analysis. The results showed the significant enrichment of DEGs in protein processing, microtubule assembly, and mitochondrial dyna­mics. Mitochondrial dyshomeostasis is a key pathological cause of disease progression, especially in female reproduction. Excessive mitophagy and imbalanced mitochondrial metabolism were shown in granulosa cells in premature ovarian insufficiency (Shang et al., 2025). Enhancement of mitophagy activity and mitochondrial function helped to recover oocyte quality in aged mice (Zhang et al., 2023). Similarly, in our study, abnormal mitochondria distribution, reduced ATP, and affected pS616-DRP1/FIS1 in oocytes indicated mitochondria damage after circadian disruption. These mitochondrial defects subsequently impaired associated organelles, including the ER and Golgi apparatus. Since mitochondrial ATP deficiency disrupts spindle assembly and chromosome segregation in mouse oocytes (Zhang et al., 2006). Aberrant ROS levels, ATP production, and Golgi apparatus distribution were also restored when light-­exposed oocytes were treated with MitoQ. We demonstrated that the abnormal subcellular activities in oocyte after circadian disruption are probably caused by affected mitochondrial homeostasis and dynamics.

For the mechanism underlying mitochondrial dysfunction, our KEGG analysis showed significant enrichment of DEGs in the AKT signaling pathway, and its reduced activity with increased PTEN was further confirmed. However, the p-AKT/AKT ratio was unchanged, indicating the decreased p-AKT was mainly due to decreased AKT level, and a compensatory mechanism of AKT phosphorylation may exist in light-exposed oocytes. Additionally, supplementation with the specific AKT activator SC79 rescued oocyte maturation, accompanied with restored mitochondrial functions and cytoskeleton organization. These findings indicated that PTEN/AKT pathway may mediate mouse fertility damage after circadian disruption. AKT, primarily localized along microtubules, is required for spindle assembly and mitochondrial homeostasis (Xie et al., 2022). AKT pathway disorder is also seen in the PCOS models induced by a high-fat diet (Nteeba et al., 2013). Considering the high correlation of occupational light exposure with PCOS, we proposed that the mechanism of AKT activity was the pathophysiology of additional light exposure-related circadian disruption in human and animal reproduction, and melatonin may reverse these defects through its endocrine regulatory and antioxidant activities. A close relationship between melatonin and AKT pathway is also reported in the steroidogenesis of bovine theca cells and ovarian aging delay (Wang et al., 2019; Yang et al., 2021), suggesting positive roles of melatonin in female reproduction and melatonin-AKT axis may present a promising therapeutic strategy to mitigate circadian rhythm-related reproductive disorders.

In summary, we identified that circadian rhythm disruption by constant light exposure induced fertility decline in the mouse model, which could be restored by melatonin supplementation. The underlying mechanism involved mitochondria dysfunction through PTEN/AKT pathway in light-exposed oocytes, further leading to impaired oocyte quality and fertilization capacity. These findings first provide a molecular basis for understanding how circadian disruption affects female reproduction, and offer a therapeutic target to ameliorate reproductive damage from circadian disruption.

## Supplementary Material

pwaf080_Supplementary_Data

## Data Availability

The data that support the findings of this study are available from the corresponding author upon reasonable request.

## Footnotes

This work was supported by the National Natural Science Foundation of China (32571001), the Fundamental Research Funds for the Central Universities of China (KJJQ2025001, RENCAI2025035), and the National Key Research and Development Program of China (2023YFD1300502). The authors declare no conflict of interest.

This study has been approved by the committee of Animal Ethics and Farewell in Nanjing Agriculture University (NJAU.No20231207186). All institutional and national guidelines for the care and use of laboratory animals were followed.

S.C.S. and P.S.L. conceived the study. P.S.L., K.H.Z., and S.L.W. performed the experiments. P.S.L. analyzed the data. R.J.M., Y.J.Z., J.Q.J., H.L.Z., and Y.W. contributed to materials and agents. P.S.L. and S.C.S. wrote the manuscript.
