# Supplementary material for: Circadian rhythm disruption declines oocyte quality for fertility via PTEN/AKT pathway
Source: Protein Cell. 2025 Sep 24;17(3):268–73. doi: 10.1093/procel/pwaf080 (PMC12987567; doi:10.1093/procel/pwaf080)
Supplement: pwaf080_Supplementary_Data [file pwaf080_supplementary_data.zip › pwaf080_Supplementary_Data/Supplementary_Materials_9.docx.16_R_clean]

**Supplementary Materials**

**Materials and Methods**

**Ethics statement and animals**

This study has been approved by the committee of Animal Ethics and Farewell in Nanjing Agriculture University (NJAU.No20231207186). All operations on mice were followed the guidelines of the Animal Research Committee of Nanjing Agriculture University, China. 10-week-old Institute of Cancer Research (ICR) male mice used in this study were obtained from Qinglongshan Animal Farm, while 4-week-old ICR female mice were from Comparative Medical Center of Yangzhou University. All mice were maintained in a standard light/dark cycle for >1 week acclimation. For female mice, the animals were randomly assigned to different groups (30 mice per group with similar mean body weight): Group control: the mice were kept under a 12 h light (daylight)/12 h darkness (0 lux) photoperiod (L/D, 7:00 am to 7:00 pm); Group light: the mice were placed under 12 h light (daylight)/12 h (300 lux) photoperiod; Group light + MT: the same as group light but giving water supplemented with 30 mg/Kg BW/day of melatonin (MT) for rescue. Light nocturnal exposure was provided by light-emitting diode which remained on all the time. Light intensity was measured on a horizontal surface in the cage with a digital illuminometer. All the light exposure protocols were performed in accordance with previous studies (Cos et al., 2006; Guan et al., 2023). After 2 weeks, all the animals were kept under a standard L/D cycle with normal daily drinking water. Body weight was measured weekly.

**Antibodies and chemicals**

Rabbit anti-Rab10 antibody (ab104859) and rabbit anti-PTEN antibody (ab32199) were from Abcam. Rabbit anti-tubulin antibody (11224-1-AP), rabbit anti-GAPDH antibody (10494-1-AP), rabbit anti-GRP78 antibody (11587-1-AP), rabbit anti-AKT antibody (10176-2-AP), rabbit anti-pSer473-AKT antibody (4060T), and rabbit anti-FIS1 antibody (10956-1-AP) were from Proteintech. Rabbit anti-pS616-DRP1 antibody (3455S) was from Cell Signaling Technology. Rhodamine-phalloidin (FHDR1) was from Cytoskeleton. Dichlorodihydrofluorescein diacetate (DCFH-DA) Kit (S0033S-1). Mitochondrial Membrane Potential Assay Kit with TMRE (C2001S), ER-tracker (C1042S-1), Golgi-tracker (C1045S-1), Horseradish peroxidase-conjugated goat anti-rabbit/mouse IgG antibodies (A0208/A0216), Wright–Giemsa staining (C0131), and Hoechst 33342 for live cells (C1027) were from Beyotime. Mito-tracker (M7512) and Hoechst 33342 for dead cells (H3570) were from Life Technologies. Lens Culinaris Agglutinin (LCA)-FITC (L32475) was from Invitrogen. Alexa Fluor 594 and 488 goat anti-rabbit antibody (ZF-0316; ZF-0511), Alexa Fluor 594 and 488 goat anti-mouse antibody (ZF-0313; ZF-0512) were from Zhongshan Golden Bridge Biotechnology. Mitoquinone mesylate (HY-100116A) and SC79 (HY-18749) were from MedChemExpress. Mouse monoclonal anti-α-tubulin-FITC antibody (F2168), melatonin (M5250), and all other unstated chemicals were from Sigma.

**Melatonin supplement**

The dosage of melatonin used was selected based on published literature (Lan et al., 2018; Xing et al., 2021). The melatonin was dissolved in ethanol and then diluted by drinking water. The final concentration of melatonin was 30 mg/Kg/day and the ethanol in drinking water was below 0.2%, which was below the safe valve from the previous reports (Cebral et al., 1997; Cebral et al., 2011). The drinking fluid was kept in standard bottles covered with aluminum foil to prevent light-induced melatonin degradation. Fresh melatonin and vehicle solutions were prepared every three days and the melatonin content was adjusted to the mean body weight of rescue group for 2 consecutive weeks.

**Fertility testing and estrous cycle evaluation**

Mice after light exposure treatment were mated with male ICR mice with proven fertility. Offspring from all the groups were kept in a standard L/D cycle, and their body weight and survival rate were measured per week from postnatal day 0 to 21/28. Mice underwent daily vaginal smear at 9:00 am for 10 consecutive days following light exposure treatment. The vaginal lavage was performed by flushing the vagina with 20 μL of saline water for 4-5 times using the same tip. Then the fluid was dispersed onto a glass slide and stained using Wright-Giemsa. The stages of proestrus (P), estrus (E), metestrus (M), and diestrus (D) were assessed based on the cell types in vaginal smears as described previously (McLean et al., 2012).

**Oocyte collection and *in vitro* maturation**

Mice were sacrificed by neck amputation after light exposure treatment. Ovaries were first collected for ovary index calculation and then denuded germinal vesicle stage oocytes (GV) were obtained with a prefabricated glass tube. Three washes with M2 medium and finally oocytes were cultured in M16 medium, sealed with paraffin oil at a 5% CO2 and 37 ℃ atmosphere for 2 h (germinal vesicle breakdown, GVBD), 9 h (metaphase Ⅰ, MⅠ), and 12 h (metaphase Ⅱ, MⅡ).

***In vivo* fertilization evaluation**

Mice after light exposure treatment were superovulated by injection of 10 IU PMSG, followed 48 h later by the injection of 10 IU hCG, and then mated with male mice. Cumulus-oocytes complexes (COCs) were collected by flushing oviducts with M2 medium 18-20 h after hCG injection, following 1 mg/mL hyaluronidase treatment for 5 min to remove cumulus cells and three washes with M2 medium. The fertilization rate was calculated.

**RNA-seq and bioinformatics analysis**

A total of 80 MⅠ stage oocytes were respectively collected for control, light, and light+MT groups. The transcriptomic analysis was performed by the Beijing Geek Gene Technology Co. Ltd. The total RNA was extracted using RNeasy Micro Kit (QIAGEN, 74004, Quigen, Toronto, Canada) and quality control was evaluated by the Qubit RNA Assay Kit (Invitrogen, Eugene, OR, USA). Low-quality reads and the linker sequence were filtered in the original RNA-seq reads by Trimmomatic (v0.39) and then the Clean Reads were aligned to the *mm10* reference genome using STAR (2.7.5c). Gene expression was calculated with featureCounts (v1.6.5) and normalized FPKM values were obtained from StringTie (v2.1.7). The significance analysis (1.2-fold change and *P* adjust < 0.05) of the results were used to identify genes that were upregulated or downregulated after light exposure. Venn diagram, Volcano plot, Gene Ontology (GO) analysis, Kyoto Encyclopedia of Genes and Genomes (KEGG) pathways, GO Chord Diagram, and Gene Set Enrichment Analysis (GSEA) were performed with the background of all the transcriptome results. The data of all sample genes were calculated according to the normalized FPKMs. Each group was repeated 3 times.

**MitoQ and SC79 treatment of oocytes**

Mitoquinone mesylate (MitoQ) and SC79 were respectively dissolved in dimethyl sulfoxide (DMSO) to a 50 mM reserve solution. 50 mM Mito-Q and SC79 were first diluted to a median concentration of 100 μM and then prepared the final concentrations of 0.1 μM and 10 μM with M16 medium. The final DMSO concentration administered to oocytes was less than 0.2‰. For *in vitro* rescue treatments, the oocytes from light exposure mice were transferred to M16 containing MitoQ or SC79 after 4 h normal culture in M16 medium.

**Immunofluorescence staining and confocal microscopy**

Denuded oocytes were fixed in 4% paraformaldehyde (PFA) for 30 min, followed by permeabilization with 0.5% Triton X-100 for 20 min and blocking in 1% bovine serum albumin (BSA)-supplemented phosphate buffer saline (PBS) for 1 h at room temperature. Then the samples were incubated with primary antibodies at 4 ℃ overnight and subsequently three washes in washing buffer before labeled with the corresponding secondary antibodies for 1 h at room temperature. The primary antibodies used were anti-α-tubulin-FITC (1:400), anti-GRP78 (1:200), anti-Rab10 (1:400), anti-Rab11a (1:500), anti-pSer616-DRP1 (1:100), and anti-pS473-AKT (1:600). The secondary antibodies used were Alexa Fluor 488/594 goat anti-rabbit (1:200), and Alexa Fluor 488/594 goat anti-mouse (1:200). For actin staining, samples were incubated with Rhodamine-phalloidin (1:200) for 1 h at room temperature. Three washes in washing buffer after that, the samples were stained with Hoechst 33342 (1:10,000) for 10 min at room temperature and mounted on glass slides for examination by a confocal laser scanning microscope (Zeiss LSM 800 META, Jena, Germany). Fluorescence results were analyzed using ZEN lite software 2012.

For cortical granule, the zona pellucida of MⅡ stage oocytes was first removed by 10 mg/mL pronase (in M2 medium) for 5 min and the samples were fixed in PFA. Three washes in PBS containing 0.3% BSA, 100 mM glycine for 15 min, followed by permeabilization with 0.1% Triton X-100 for 5 min. Two washes in PBS for 5 min and then incubated with lens culinaris agglutinin (LCA)-FITC staining (1:100; in PBS) for 1 h at room temperature, subsequently washed in 0.3% BSA, 0.01% Triton X-100 in PBS for 15 min. DNA was stained with Hoechst 33342 (1:10,000) for 10 min at room temperature. Finally, the samples were mounted on glass slides and imaged as described above.

**Reactive oxygen species (ROS) detection**

Oxidation-sensitive fluorescent probe DCFH-DA Kit was used to analyze the ROS level. The samples were transferred from M16 to DCFH-DA (1:800, with M16 medium dilution) for 30 min at 37 ℃ and 5% CO2. Then, three washes with M2 medium and then scanned the live oocytes with a laser confocal microscope by Zeiss LSM 800 META.

**Detection of mitochondria, ER,** **MMP, and Golgi apparatus**

The distribution of mitochondria and ER in living oocytes was respectively detected by Mito-Tracker Red (1:600) and ER-Tracker Green (1:400) with M16 medium for 30 min at 37 ℃ and 5% CO2. Mitochondrial membrane potential detection was performed using a Mitochondrial Membrane Potential (MMP) Assay Kit with TMRE (1:600) with M16 medium for 30 min at 37 ℃ and 5% CO2. For Golgi apparatus detection, oocytes were first incubated with 10 mg/mL pronase (in M2 medium) for 5 min to remove the zona pellucida. Then the living oocytes were incubated with Golgi-Tracker Red (1:100) in an M16 medium for 30 min at 4 ℃. DNA was stained with Hoechst 33342 (1:100) for 30 min. Finally, three washes with M2 medium and then scanned the live oocytes with a laser confocal microscope by Zeiss LSM 800 META.

**Fluorescence intensity analysis**

Image J software (National Institutes of Health, Bethesda, MD, USA) was used for fluorescence intensity measurement. The samples were placed in different area on the same glass slide and scanned in the same environment using the same parameters. A region of interest on the target image was analyzed and the average of all mean values in each group were used to perform statistical analysis. The fluorescence intensity of the control group was recognized as 1. For abnormal ROS intensity calculations, the oocyte whose intensity quantified by Image J was above the average of all mean values plus SEM in each group was considered abnormal intensity of oocyte.

**RNA extraction and quantitative real time**

Total RNA was extracted from approximately 30 MⅠ stage oocytes using a Dynabeads mRNA DIRECT™ Kit (Invitrogen, Dynal, Oslo, Norway), followed by cDNA synthesis according to cDNA synthesis kit (Takara Biomedical Technology, Dalian, China) and stored at -20 °C. Each real time-PCR reaction system consisted of 10 µL of ChamQ Universal SYBR qPCR Master Mix (Q711, Vazyme, Nanjing, China), 1.6 µL of specific primers, 6.4 µL of pure water, and 2 µL cDNA sample. The relative quantitative analysis of genes was performed by QuantStudio 5 (Applied Biosystems, Carlsbad, CA, USA) and normalized to the corresponding *Gapdh* with the 2^─△△CT^. The primers used were listed in Table S1.

**Mitochondrial DNA copy number analysis**

Thirty MⅠ stage oocytes from each group were extracted using a DNA extracted Kit (Sangon, Shanghai, China). Mitochondrial DNA (mtDNA) copy number was quantified by q-PCR and normalized to the corresponding *18s* with the 2^─△△CT^ method. The mouse mtDNA specific primers were listed in Table S1.

**ATP content detection**

Adenosine 5′-triphosphate (ATP) bioluminescent somatic cell assay kit (FLASC, Sigma) was used to quantify the total ATP content in oocytes. Thirty MⅠ stage oocytes were diluted with ATP releasing reagent in pure water on ice. The released sample was mixed with the ATP assay mix containing luciferase and the relative content of ATP was obtained by a multimode microplate reader (TecanSpark) based on bioluminescence value.

**Western blot analysis**

Approximately 150 living oocytes per group were lysed with NuPAGE LDS Sample Buffer and boiled at 100 ℃ for 10 min and stored at -20 ℃. The proteins were separated by electrophoresis on 4-20% SurePAGE (M00656, Nanjing GenScript Biotech Co., Nanjing City, China) at 140 V for 1 h, followed by transferring onto polyvinylidene fluoride membranes (Millipore, Billerica, MA, USA) with eBlot^TM^ L1 (L00686C, Nanjing GenScript Biotech Co., Nanjing City, China). Then the membranes were blocked with TBST containing 5% nonfat milk for 4 h at 4 ℃ and subsequently incubated with anti-β-actin (1:1,000), anti-GAPDH (1:1,000), anti-tubulin (1:2,000), anti-GRP78 (1:1,000), anti-pSer616-DRP1 (1:1,000), anti-FIS1 (1:1,000), anti-AKT (1:1,000), anti-pS473-AKT (1:1,000), and anti-PTEN (1:1,000) at 4 ℃ overnight. After three washes in TBST, immunoblots were labeled with conjugated anti-mouse or anti-rabbit antibodies (1:2,000) for 1 h at room temperature. Finally, three washes in TBST were performed and the membranes were processed using the ECL Plus Western Blotting Detection System (Tanon-3900), and then Image J software was used to analyze the band intensity value. The Restore PLUS Western Blot Stripping Buffer was used to remove the primary and secondary antibodies from a Western blot so that the blot could be re-probed.

**Statistical analysis**

All experiments included at least three biological replicates. Statistical analyses were performed using the GraphPad Prism 9.5.0 software (GraphPad, San Diego, CA). Normality and variance homogeneity tests were assessed first. Parametric and homogeneous data were analyzed by ordinary one-way ANOVA with Dunnett’s multiple comparisons test. Non-parametric data were evaluated by Kruskal-Wallis test followed by Dunn’s test, while heterogeneous data employed Welch’s ANOVA with Dunnett’s T3 test. For Western blot, mRNA/mt-DNA, and fluorescence intensity analysis, to minimize technical variability between replicates, all control groups were set as “1” and treatment groups were normalized accordingly. The lack of variance in the control group precluded standard group comparisons. Therefore, tests against the control utilized a one-sample *t*-test (parametric) or one-sample Wilcoxon test (non-parametric) against the value "1". Comparisons among treatment groups involved unpaired t-tests or one-way ANOVA, with Mann-Whitney U tests for non-parametric data and Welch's correction for unequal variances. Offspring survival was compared using the Log-rank test with Bonferroni correction. The number of mice/oocytes observed (n) was put in parentheses. The *P*-value < 0.05 was considered statistically significant. *, indicates the significance between control and other groups. #, indicates the significance between the light and light+MT groups.

**References**

Cebral, E., Abrevaya, X.C., and Mudry, M.D. (2011). Male and female reproductive toxicity induced by sub-chronic ethanol exposure in CF-1 mice. Cell Biol Toxicol 27, 237-248.

Cebral, E., Lasserre, A., Rettori, V., and De Gimeno, M.A. (1997). Impaired mouse fertilization by low chronic alcohol treatment. Alcohol Alcohol 32, 563-572.

Cos, S., Mediavilla, D., Martínez-Campa, C., González, A., Alonso-González, C., and Sánchez-Barceló, E.J. (2006). Exposure to light-at-night increases the growth of DMBA-induced mammary adenocarcinomas in rats. Cancer Lett 235, 266-271.

Guan, Y., Xu, M., Zhang, Z., Liu, C., Zhou, J., Lin, F., Fang, J., Zhang, Y., Yue, Q., Zhen, X., et al. (2023). Maternal circadian disruption before pregnancy impairs the ovarian function of female offspring in mice. Sci Total Environ 864, 161161.

Lan, M., Han, J., Pan, M.H., Wan, X., Pan, Z.N., and Sun, S.C. (2018). Melatonin protects against defects induced by deoxynivalenol during mouse oocyte maturation. J Pineal Res 65, e12477.

McLean, A.C., Valenzuela, N., Fai, S., and Bennett, S.A. (2012). Performing vaginal lavage, crystal violet staining, and vaginal cytological evaluation for mouse estrous cycle staging identification. J Vis Exp, e4389.

Xing, C.H., Wang, Y., Liu, J.C., Pan, Z.N., Zhang, H.L., Sun, S.C., and Zhang, Y. (2021). Melatonin reverses mitochondria dysfunction and oxidative stress-induced apoptosis of Sudan I-exposed mouse oocytes. Ecotoxicol Environ Saf 225, 112783.

**Supplementary Figure legends**

**Fig. S1. Constant light exposure affects mouse estrous cycle, offspring survival, and circadian gene expression.** (A) Body weight during and after 2 weeks of light treatment. (B) Representative images of proestrus, estrus, metestrus, and diestrus in the mouse estrus cycle. P: proestrus, E: estrus, M: metestrus, D: diestrus. Bar = 50 μm. Estrous cycle curves of three mice in the control, light, and light+MT groups, respectively. (C) Average estrous cycle duration in control (n=10), light (n=10), and light+MT (n=10) female mice. (D) The survival rate of litters in control, light, light+MT mice from postnatal day 0 to 28. (E) Body weight of litters in control, light, light+MT mice from postnatal day 0 to 21. (F) Representative images and ovary/body weight ratio of ovary in control (n=42), light (n=46), light+MT (n=47) mice. Bar= 3 mm. (G) Representative images of GV stage oocyte and its number in control (n=11), light (n=11), light+MT (n=11) mice ovary. Bar = 200 μm. (H) The mRNA level of *Clock*, *Csnk1e*, and *Bmal1* in control, light, and light+MT oocytes. (I) The percentage of oocyte that underwent GVBD in control (n=116), light (n=145), and light+MT (n=148) mouse was quantified. *, indicates the significance between the control and light groups. #, indicates the significance between the light and light+MT groups. Data are presented as mean ± SEM of three biological repeats. ns, *P*>0.05. *, *P*<0.05. **, *P*<0.01. #, *P*<0.05. ##, *P*<0.01.

**Fig. S2. Constant light exposure disturbs global mRNA expression level in mouse oocytes.** (A) The volcano plot analysis of DEGs (downregulated, blue; upregulated, red) among control, light, and light+MT group oocytes. (B) GO enrichment analysis of DEGs between light and control group oocytes. (C) GO enrichment analysis of DEGs between light+MT and light group oocytes. (D) KEGG enrichment analysis of DEGs between light and control oocytes. (E) KEGG enrichment analysis of DEGs between light+MT and control oocytes. (F) GO Chord Diagram depicting the relationship between DEGs and GO pathways of ribosome, microtubule, chromosome segregation, ATP hydrolysis, and DNA repair. (G) GO Chord Diagram depicting the relationship between DEGs and mitochondria-related GO pathways.

**Fig. S3.** **Constant light exposure impairs mitochondrial functions and dynamics in mouse oocytes.** (A) The level of mitochondrial DNA copy number in control, light, and light+MT oocytes. (B) The level of ATP in the MⅠ stage of control, light, and light+MT groups. (C) The bubble graph of organelles-related GO pathways based on the DEGs between control and light groups. (D) Representative images and abnormal ROS intensity in MⅠ stage oocyte from control (n=46), light (n=57), and light+MitoQ (n=38) groups. The intensity of ROS that was above mean + SEM was considered abnormal. Green, ROS. Bar = 80 μm. (E) The ATP level in the MⅠ stage of control, light, light+MT, light+MitoQ, and light+SC79 groups. (F) Representative images and abnormal percentage of Golgi apparatus in oocyte from control (n=50), light (n=47), and light+MitoQ (n=55) groups. Red, Golgi-tracker. Blue, DNA. Bar = 20 μm. *, indicates the significance among control, light, light+MitoQ, and light+SC79 groups. #, indicates the significance between the light and light+MT groups. Data are presented as mean ± SEM of three biological repeats. *, *P*<0.05. **, *P*<0.01. #, *P*<0.05. ##, *P*<0.01.

**Fig. S4.** **Constant light exposure induces abnormal ER and Golgi apparatus functions in mouse oocytes.** (A) Representative images and relative intensity of GRP78 in oocyte of control (n=37), light (n=34), and light+MT (n=35) groups. Red, GRP78. Blue, DNA. Bar = 20 μm. (B) Representative images of Rab10 in oocyte from control, light, and light+MT groups. Green, Rab10. Blue, DNA. Bar = 20 μm. The arrows highlight the Rab10 signal in the subcortical regions. Abnormal distribution rate and relative intensity of cytoplasmic Rab10 in the oocytes from control (n=52), light (n=52), and light+MT (n=47) groups were quantified. (C) Representative images of Rab11a in oocyte from control, light, and light+MT groups. Cyan, Rab11a. Blue, DNA. Bar = 20 μm. The arrows highlight the Rab11a signal in the subcortical regions. Abnormal distribution rate and relative cytoplasmic intensity of Rab11a in the oocytes from control (n=38), light (n=39), and light+MT (n=37) groups were quantified. (D) Representative images and abnormal cortical granule (CG) distribution rate in oocyte from the control (n=49), light (n=53), and light+MT (n=49) groups. Black, cortical granule (CG). Blue, DNA. Bar = 20 μm. The arrows highlight the CG-free domain (CGFD) near chromosomes. Data are presented as mean ± SEM of three biological repeats. *, indicates the significance between the control and light groups. #, indicates the significance between the light and light+MT groups. Data are presented as mean ± SEM of three biological repeats. *, *P*<0.05. **, *P*<0.01. #, *P*<0.05. ###, *P*<0.001.

**Table S1. The specific primers used in our study**

| Gene | Forward primer | Reverse primer |
| --- | --- | --- |
| *Clock* | CTTCCTGGTAACGCGAGAAAG | GTCGAATCTCACTAGCATCTGAC |
| *Csnk1e* | AAGCTCGAATGTGTGAAGACG | TGACCATCACGTTATAGTCTCCC |
| *Bmal1* | TGACCCTCATGGAAGGTTAGAA | GGACATTGCATTGCATGTTGG |
| *18s* | CGCGGTTCTATTTTGTTGGT | AGTCGGCATCGTTTATGGTC |
| *16s* | CTAAAGTTTAACGGCCGCGG | CCTCGTTTAGCCGTTCATGC |
| *Gapdh* | AGGTCGGTGTGAACGGATTTG | GGGGTCGTTGATGGCAACA |
